# Self-recordings of upper arm elevation during cleaning – comparison between analyses using a simplified reference posture and a standard reference posture

**DOI:** 10.1186/s12891-018-2328-8

**Published:** 2018-11-15

**Authors:** Camilla Dahlqvist, Catarina Nordander, Mikael Forsman, Henrik Enquist

**Affiliations:** 1 Division of Occupational and Environmental Medicine, Department of Laboratory Medicine, Lund University, SE-221 85 Lund, Sweden; 2 Division of Ergonomics, KTH Royal Institute of Technology, SE-141 57 Huddinge, Sweden

**Keywords:** Inclinometry, Zero position, Self-measurement, Physical workload, Angular velocity, Arm elevation, Hotel housekeeping

## Abstract

**Background:**

To reduce ergonomic risk factors in terms of awkward and constrained postures and high velocities, it is important to perform adequate risk assessments. Technical methods provide objective measures of physical workload. These methods have so far mainly been used by researchers. However, if written instructions how to apply the sensors and how to adopt the reference posture are provided, together with triaxial accelerometers, it may be possible for employees to record their own physical workload. The exposure in terms of e.g. upper arm elevations could then easily be assessed for all workers in a workplace. The main aims of this study were: 1) to compare analyses for self-recording of upper arm elevation during work using a simplified reference posture versus using a standard reference posture, and 2) to compare the two reference postures.

**Methods:**

Twenty-eight cleaners attached an accelerometer to their dominant upper arm and adopted a simplified reference according to a written instruction. They were thereafter instructed by a researcher to adopt a standard reference. Upper arm elevations were recorded for 2 or 3 days. Each recording was analysed twice; relative to the simplified reference posture and relative to the standard reference posture. The group means of the differences in recorded upper arm elevations between simplified and standard reference analyses were assessed using Wilcoxon signed ranks test. Furthermore, we calculated the group mean of the differences between the simplified reference posture and the standard reference posture.

**Results:**

For arm elevation during work (50^th^ percentile), the group mean of the differences between the two analyses was 0.2° (range -7 – 10°). The group mean of the differences between the two references was 9° (range 1 – 21°). The subjects were able to follow the instructions in the protocol and performed self-recording of upper arm elevation and velocity.

**Conclusions:**

The small difference between the two analyses indicates that recordings performed by employees themselves are comparable, on a group level, with those performed by researchers. Self-recordings in combination with action levels would provide employers with a method for risk assessment as a solid basis for prevention of work-related musculoskeletal disorders.

**Electronic supplementary material:**

The online version of this article (10.1186/s12891-018-2328-8) contains supplementary material, which is available to authorized users.

## Background

Many jobs involve repetitive work, prolonged muscular load and work performed in awkward and constrained postures. Such work are known to be risk factors for developing work-related musculoskeletal disorders (WMSDs) in the neck/shoulder region, arms, and hands [1–4]. To reduce these risks, it is important to perform risk assessments, and to implement organisational and technical measures when necessary [5]. The reliability of risk assessments is important as this affects the decisions made and the priorities afforded different interventions [6].

Several kinds of risk assessment methods are available, such as self-reporting, observational methods and technical methods, all of which have advantages and disadvantages [7]. For example, in self-reporting, which has the advantage of being practical in large groups, overestimation of the workload is common among individuals with pain [8]. Observational methods are often easy to use and interpret, and give a rough estimate of postures during work, but results vary between observers [9]. As observational methods have no common references, they tend to give different results when assessing the risk of developing WMSDs [10, 11]. Technical methods, on the other hand, provide exact numerical values for both postures and movements during work, i.e. upper arm elevation and velocity [12].

There is a commonly held belief that technical methods require expensive equipment, technical understanding and are time-consuming [13]. However, low-cost sensors for recording of elevations and velocities during work are now commercially available [14, 15]. These sensors have also made it feasible to measure the workload over several days [16]. Many studies have been performed previously in which the workload on a few individuals has been recorded during 1 day [17, 18]. With the advent of low-cost sensors, it is now possible to monitor the entire workforce over several days.

Measurements over extended periods of time are important in planning job rotation as a measure for the prevention of WMSDs [19]. Furthermore, measurements made over several days will give a better idea of the loads experienced on an average working day [20]. Such an average measurement is likely to be more strongly correlated with the prevalence of WMSDs than those from one-day recordings. So far, the number of technical recordings has been limited, mainly due to the need for researchers.

If self-recording of physical workload was possible, all the employees’ workload at almost any workplace could be explored for several days. Such recordings would be invaluable when performing risk assessments. However, it would be necessary to develop easily understandable instructions so that the employees can attach the equipment and calibrate it, i.e. adopt a reference posture. The reference posture should have a high reproducibility, and this can be studied if recordings are performed over several days. The reference posture should also be easy to adopt, and without the need of extra material. Such a reference would rule out our standard reference posture, which we have used in many studies, as the latter requires a chair and a dumbbell [17, 21–23]. A self-recording method also requires a reliable method of identifying the reference, as this defines 0 degrees of inclination. Furthermore, the starting and stopping times of work and breaks should be noted, to distinguish between working time and leisure time.

One occupation with a high physical workload and a high risk of WMSDs is cleaning [24]. As an example, the prevalence of complaints and diagnoses in neck/shoulders has been reported to be 48% among female hospital cleaners working in a traditional work organisation [25]. Around the world there are many employees working as cleaners and it is important to perform risk assessments of their work in a cost-effective manner. Therefore, we would like to test the self-recording method in the cleaning industry.

The main aims of this study were: 1) to compare analyses for self-recording of upper arm elevation during work using a simplified reference posture versus using a standard reference posture, and 2) to compare the two reference postures. Other aims were to study the between-day repeatability in the simplified reference posture, and to assess the suitability of a protocol for self-recording. Furthermore, we aimed to compare the physical workload, the between-day repeatability of the workload, and to assess the risk of musculoskeletal disorders among different types of cleaning.

## Subjects and methods

### Study design

This was a field study including two parts. In part one (self-recordings), workers received a protocol with instructions on how to attach a triaxial accelerometer (GC inclinometer) to the upper arm, and how to adopt a reference posture. It was adopted by the cleaners themselves, without the need of extra material, and referred to as the simplified reference posture. A researcher then instructed each of them to adopt the standard reference posture. The workers wore the GC inclinometer continuously, both day and night, for 2 or 3 days. They repeated the simplified reference posture each morning and noted starting and stopping times of work and lunch breaks for each day in a provided form.

In part two (researchers’ recordings), which was conducted on different days than the self-recordings, the researchers attached the GC inclinometer to the worker’s right upper arm and instructed each subject to perform the standard reference posture. The researchers followed each worker during the one-day recording and noted exact starting and stopping times for work and breaks.

### Subjects

#### Self-recordings

Twenty-eight subjects, 24 women and 4 men, participated in the study (Table 1). Their mean age was 43 years (range 22–58). Twenty-four of the subjects (20 women and 4 men) worked as hotel cleaners and 4 (all women) as office cleaners. Three of the 28 subjects were native Swedish speakers, while the other 25 spoke English and Swedish of varying quality. All the hotel cleaners cleaned hotel rooms (denoted hotel housekeeping). Some of them (eleven subjects) also had other tasks such as cleaning corridors, conference rooms, pool areas and/or dining rooms (denoted hotel housekeeping+). The office cleaners cleaned mainly offices, but also toilets, changing rooms, corridors and dining rooms.

#### Researchers’ recordings

Fourteen right-handed female hotel cleaners participated in standard one-day recordings performed by professionals (Table 1). Their mean age was 42 years (range 22–57). They all cleaned hotel rooms. Five of these also performed self-recording, on separate occasions.

**Table 1:** Anthropometric characteristics

	Self-recording			Researchers’ recording		
	Height (cm)	Weight (kg)	BMI	Weight (cm)	Height (kg)	BMI
	160	50	20	160	50	20
	170	73	25	170	73	25
	172	61	21	172	61	21
	–	–	–	–	–	–
	169	70	25	169	70	25
	–	82	–			
	168	65	23			
	176	73	24			
	168	66	23			
	167	54	19			
	168	80	28			
	150	42	19			
	174	106	35			
	168	80	28			
	153	50	21			
	–	–	–			
	159	82	32			
	155	59	25			
	–	–	–			
	154	50	21			
	160	50	20			
	158	62	25			
	169	74	26			
	167	65	23			
	146	51	24			
	163	63	24			
	148	48	22			
	168	75	27			
				177	69	22
				160	50	20
				165	–	–
				162	–	–
				158	63	25
				160	61	24
				160	60	23
				157	52	21
				172	65	22
Mean	163	65	24	165	61	22.5
SD	8.4	15	4	6.5	8.0	2.1

Height, weight and BMI for the 37 subjects participating in the study. Twenty-eight subjects participated in the self-recording and fourteen subjects participated in the researchers’ recording. Five subjects participated in both types of recordings

- missing data

### Materials

Triaxial accelerometers with an integrated data logger (USB Accelerometer Model X16-mini, Gulf Coast Data Concepts, LLC, Waveland, MS, USA, “GC inclinometer”) with a sampling frequency of 25 Hz were used. This frequency is sufficient as it has been shown that 99.5% of the signal power for wrist (and it is not expected to be higher for the upper arms) was contained in the 0–5 Hz band in occupational repetitive work [26]. The size was 5 × 2.4 × 1.3 cm and they contained a 2 GB memory for data logging, a female micro USB-connector and a rechargeable battery [14]. The accelerometer was attached to the upper arm, just below the insertion of the deltoid muscle, with double-sided adhesive tape and fixed with plastic film (Tegaderm™, 3 M Health Care, St Paul, MN, USA) to secure them from falling off.

### Procedures

#### Standard reference posture

**Fig. 1:**
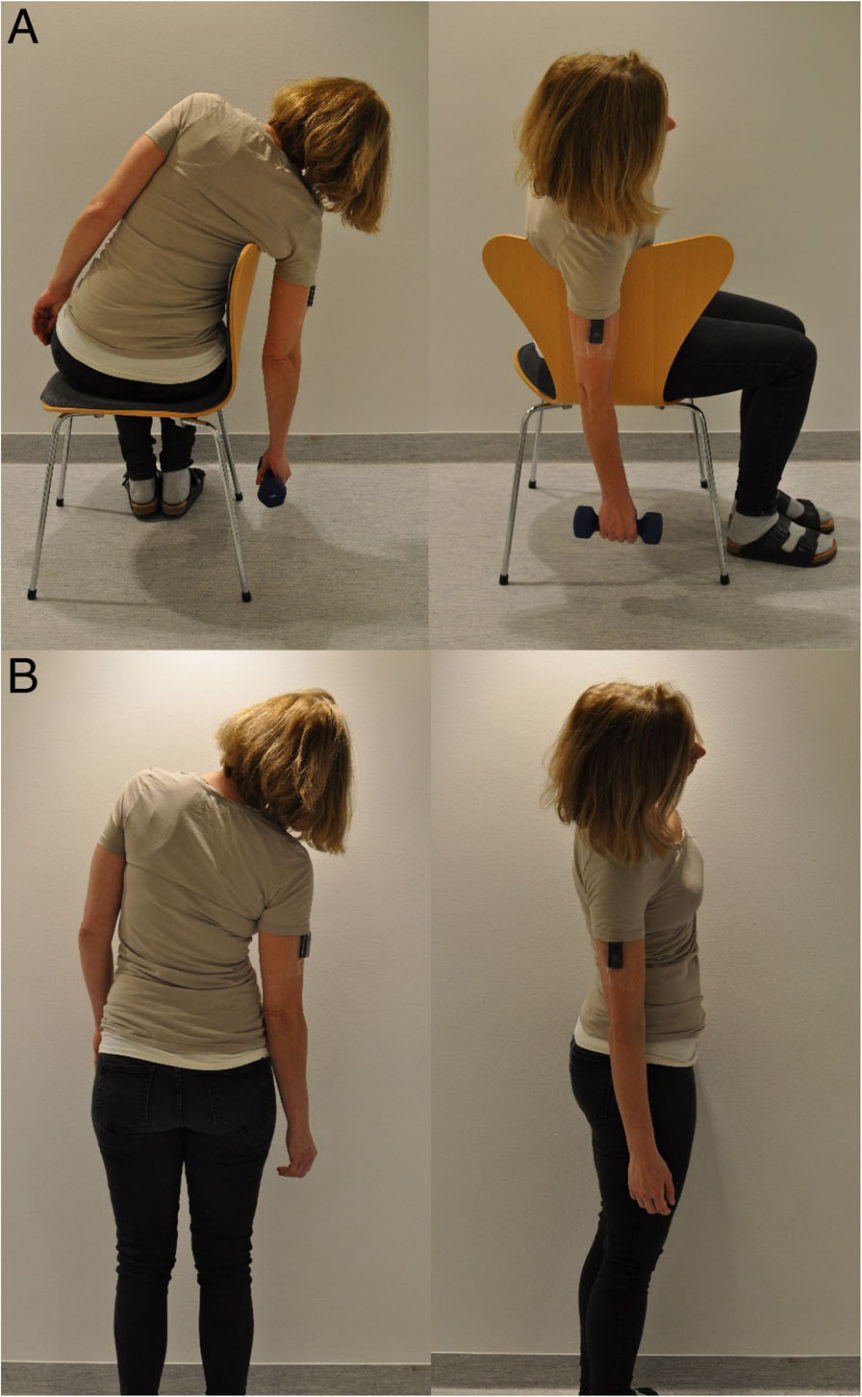
**a** The standard reference posture, and (**b**) the simplified reference posture

The researcher instructed the subject to sit on a chair and lean towards the backrest with the arm hanging vertically over the backrest, holding a dumbbell in the hand (Fig. 1a) [21].

#### Simplified reference posture

The subject followed the instructions in the protocol and leaned to the right with the arm alongside the body and with an extended elbow for about 20 s (Fig. 1b) [14].

#### The protocol

The self-recording method was tested in the cleaning industry. We made one Swedish and one English version of the protocol, as it is known to be a high proportion of immigrants among the employees [27]. Twenty-five subjects chose to use the Swedish version, while three subjects chose the English version. The protocol with instructions for using the GC inclinometer consisted mainly of pictures with short explanations how to attach the GC inclinometer and how to perform the simplified reference posture (see Additional file 1). The researcher noted that the first subjects seemed to have some difficulties in understanding the Swedish and English instructions properly, due to language barriers. Therefore, we improved the protocol in steps during the study. The first change (version 2) was to add instructions on how to start the GC inclinometer (which for the first subjects had been performed by the researcher), to obtain a complete instruction for self-recording of upper arm elevation and velocity. We also simplified the part on how to attach the inclinometer. The second change (version 3) was to add a second series of toe jumps after the simplified reference posture to improve our ability to determine which part of the recording corresponded to it. To make it easier for the subjects to perform the self-recording, minor changes were made throughout the study, such as highlighting the most important steps (starting the device and performing the simplified reference posture), numbering the various steps in the protocol and simplifying the language in the text boxes. Three versions of the protocol were used. Version 1 was used by four subjects, version 2 was used by five, and version 3 was used by 19 subjects. A few subjects needed help to start the GC inclinometer and some of them had to be reminded to adopt the simplified reference posture. However, the need for help decreased with improved versions of the protocol.

#### Self-recordings

Each subject was given a GC inclinometer and a protocol with instructions. Nineteen subjects (at twelve different times) individually followed the protocol and attached the GC inclinometer, performed five toe jumps and adopted the simplified reference posture by themselves. The toe jumps were later used to find this part of the recording. At one occasion, nine subjects were helped by their supervisor, due to lack of time. The supervisor started and attached the GC inclinometer, and instructed each subject how to perform the simplified reference posture. The supervisor had not used the protocol previously.

For each subject, the researcher did a brief visual inspection of that the GC inclinometer was attached properly. The researcher then instructed each of the subjects to perform the standard reference posture*.*

The subjects were instructed to perform the simplified reference posture every morning and to note the time for this and the starting and stopping times of work and lunch breaks in the provided form. They were instructed to apply more plastic film if needed and they were also told to remove the GC inclinometer if they experienced itching or irritation of the skin. Nineteen of the subjects wore the GC inclinometer for 3 days and nine subjects wore it for 2 days. At the end of the second or third working day, the researcher instructed the subject to perform the standard reference posture again, and then removed the GC inclinometer. In four cases the supervisor removed the GC inclinometer, and one subject removed it herself. The stop time was noted.

#### Researchers’ recordings

Researchers experienced in technical methods attached the GC inclinometer to the subject’s right upper arm, one subject at a time, on different days from the self-recordings. Each subject was instructed to adopt the standard reference posture for the right upper arm. The researchers followed each subject during their working day, noting the exact starting and stopping times for work, breaks, and different work tasks.

#### Questionnaire

To further assess the suitability of the protocol and the self-recording method, all subjects were asked, after the recording, to answer six questions about their perceptions of the self-recording.

### Data processing and analyses

The data were processed with the EMINGO software suite, developed by the Division of Occupational and Environmental Medicine in Lund, Sweden using MATLAB (version 2016b, Math Works INC., Natick, MA, USA). The data were resampled at 20 Hz, anti-aliased, low-pass filtered (5 Hz), and visually inspected.

#### Self-recording

Upper arm elevations and velocities were recorded continuously but only the data on work were analysed. Lunch breaks were excluded according to the times noted in the provided form. The data were analysed twice; once using the simplified reference posture as reference (henceforth referred to as the simplified reference analysis) and once using the standard reference as reference (henceforth referred to as the standard reference analysis). The 1^st^, 10^th^, 50^th^, 90^th^ and 99^th^ percentiles of the angular distribution (°) and the percentage of time the arm was elevated above 30°, 60° and 90° were calculated. Furthermore, the median generalised angular velocity (°/s) was derived for each subject. 1 °/s = 0.017 rad s^− 1^ and 1 rad s^− 1^ = 57.3 °/s. Group means of upper arm elevations and velocities were calculated for comparisons between the simplified reference analysis and the standard reference analysis. Further, for each subject we calculated the differences between the results derived from the two different analyses, as well as the absolute differences (i.e. the non-negative difference, regardless of sign). Then the group means of the differences and the group means of the absolute differences were calculated.

Furthermore, for each subject, we calculated the difference between the simplified reference posture and the standard reference posture (°). In most cases we used the references from day 1. In one case, the GC inclinometer fell off during day 1. The subject attached it again, and the researcher (who was still there) instructed her, during her lunch break, to perform the standard reference posture again. Another subject appeared to have replaced the GC inclinometer upside down after it had fallen off during the morning day 1 (detected during data analysis), and therefore this part of the recording was discarded. For this subject, the standard reference posture from day 3 was used. The simplified reference posture from day 2 was used for both these subjects.

The first and second simplified reference posture were used to investigate the reliability of the reference. Nine of the subjects performed the simplified reference posture on one occasion only and were therefore excluded when analysing the within-subject variation of the reference.

The within-subject variation in workload between the first and second working days was also calculated among the hotel cleaners. Then, two recordings were excluded because the subjects removed their GC inclinometer while showering after day 1. They had replaced the device after showering, but did not repeat the simplified reference posture, and therefore, the data for the remaining days had to be rejected. The remaining 22 recordings were divided into hotel housekeeping and hotel housekeeping+, with eleven subjects in each group.

When comparing upper arm elevations and velocities between the specific types of cleaning (hotel housekeeping, hotel housekeeping+, and office cleaning), as well as when comparing with the researchers’ one-day recordings of hotel housekeeping, the standard reference analysis was used. The four men were excluded from these calculations, to be able to compare them with previous and future recordings, where the results for women and men are separated [28].

#### Researchers’ recordings

Upper arm elevations and velocities during the working day were analysed, lunch breaks excluded. The same measures as for the self-recordings were calculated; the percentiles of the angular distribution (°) and the percentage of time the arm was elevated above 30°, 60° and 90° were calculated for each subject. The median generalised angular velocity (°/s) was also derived, and group means of both elevations and velocities were calculated.

#### Statistical analyses

The statistical analyses were carried out with IBM SPSS Statistics Version 22 (SPSS, Chicago, IL, USA). The alpha level was set at 0.05. Comparisons between group means of upper arm elevations for the two reference analyses were performed using Wilcoxon signed ranks test. The within-subject variation was calculated using one-way ANOVA for the simplified reference posture and for the upper arm elevations and velocities during work. The 50^th^ and 90^th^ percentiles of upper arm elevation and the median generalised angular velocity were the dependent variables, and subject was the independent variable. To investigate the repeatability of the simplified reference posture and of the workload between working days, the repeatability coefficient (°) and the intraclass correlation coefficient (ICC) were calculated [29, 30]. We used ICC (1,1) i.e. one-way random effects model, absolute agreement, single measures. ICC estimates less than 0.5, between 0.5 and 0.75, between 0.75 and 0.9, and greater than 0.90 indicate poor, moderate, good, and excellent reliability, respectively [31]. The difference between the simplified reference posture and the standard reference posture of two following occasions, respectively, as well as upper arm elevations (50^th^ and 90^th^ percentiles) and the median angular velocity of two working days were the input variables in the model. Comparisons between group means of different types of cleaning were performed using Kruskal-Wallis one-way analysis of variance. Post hoc analyses for *p*-values < 0.05 was performed using Mann-Whitney U-test. The non-parametric tests were used since the data were not normally distributed.

## Results

### Simplified reference analysis versus standard reference analysis

#### Recordings of workload

The group means of upper arm elevation and the percentage of time above 30°, 60° and 90° during work were very similar between the simplified reference analysis and the standard reference analysis (Table 2). The upper arm velocity was identical (data not shown), as this is not dependent on the reference.

**Table 2:** Group means of upper arm elevations during work for the simplified and the standard reference analyses

	Simplified reference analysis	Standard reference analysis	
	Mean (range)	Mean (range)	*p*-value
Percentile (°)			
1^st^	5 (2 – 8)	5 (2 – 10)	0.68
10^th^	14 (7 – 21)	13 (8 – 20)	0.98
50^th^	30 (20 – 47)	30 (22 – 38)	0.98
90^th^	64 (45 – 91)	64 (50 – 86)	0.95
99^th^	109 (88 – 132)	110 (94 – 134)	0.30
Percentage of time			
> 30°	49 (27 – 78)	49 (34 – 63)	0.95
> 60°	13 (4 – 35)	12 (5 – 24)	0.95
> 90°	4 (1 – 10)	3 (1 – 8)	0.98

Group means (°) for the simplified reference analysis and the standard reference analysis at the 1^st^, 10^th^, 50^th^, 90^th^ and 99^th^ percentiles of upper arm elevation and the percentage of time above 30°, 60° and 90° for the 28 subjects during work. *P*-values for difference calculated with Wilcoxon signed rank tests

**Fig. 2:**
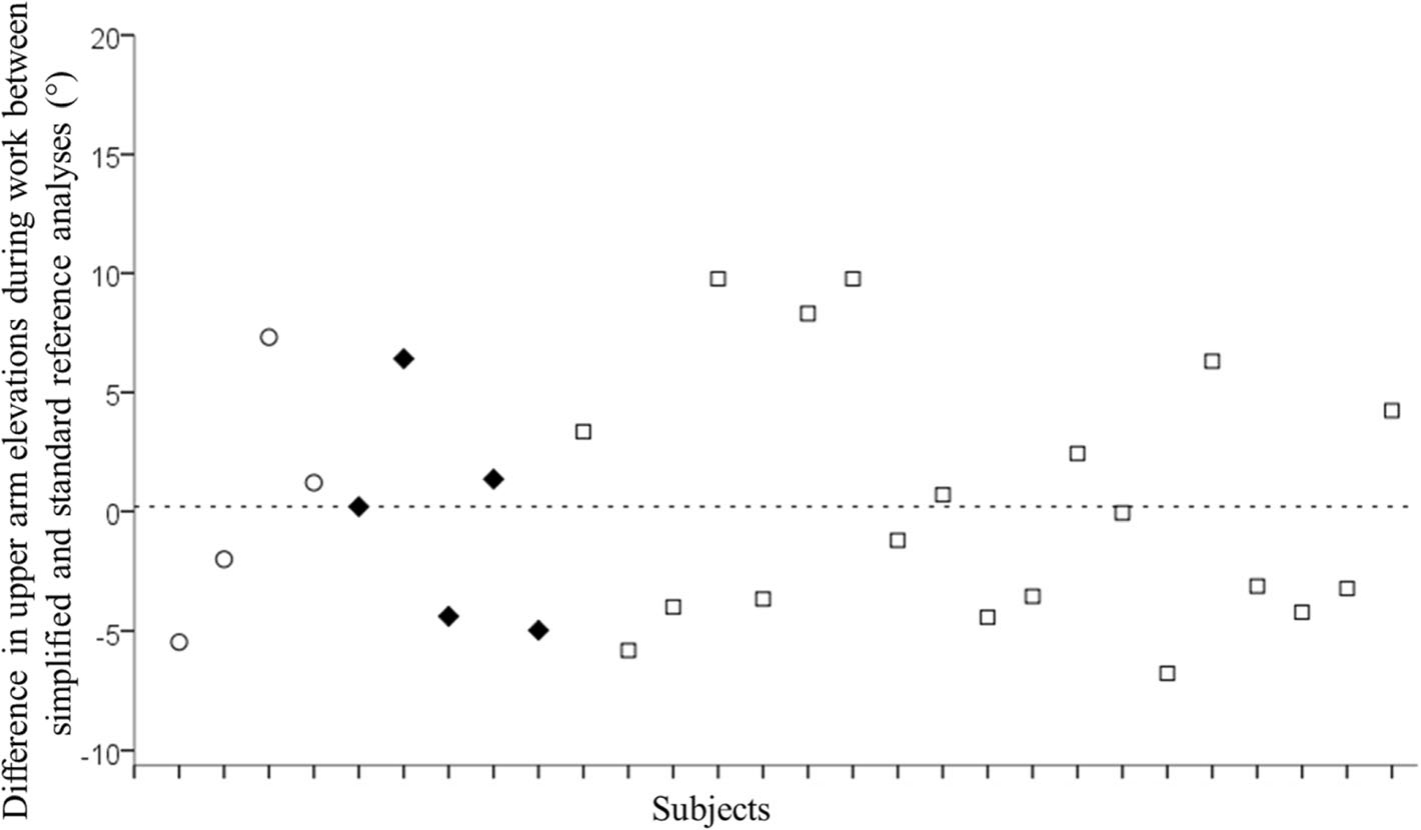
Individual difference (°) between the simplified reference analysis and the standard reference analysis from day 1 at the 50^th^ percentile of upper arm elevation during work for the 28 subjects. The dashed line indicates the group mean difference (0.2°). Version 1 of the self-recording protocol was used by four subjects (**○**), version 2 was used by five (◆) and version 3 was used by 19 subjects (□)

The individual differences between the simplified reference analysis and the standard reference analysis at the 50^th^ percentile of arm elevation during work are shown in Fig. 2. The group mean difference was 0.2° (range -7 – 10°).

The group mean of the absolute differences in the 50^th^ percentile of arm elevation was 4° (range 0 – 10°; Table 3), and for the percentage of time above 30° it was 9% (range 0 – 21%), for 60° 2% (0 – 11%), and for 90° 1% (0 – 3%).

**Table 3:** Group means of the absolute differences of upper arm elevations during work between the simplified and the standard reference analyses

	Mean absolute difference (range)
Percentile (°)	
1^st^	1.8 (0.0 – 4.4)
10^th^	3.8 (0.2 – 9.1)
50^th^	4.2 (0.1 – 9.8)
90^th^	4.2 (0.0 – 12)
99^th^	4.7 (0.3 – 18)
Percentage of time	
> 30°	9.3 (0.2 – 21)
> 60°	2.3 (0.0 – 11)
> 90°	0.6 (0.1 – 2.9)

The group mean of the absolute differences (°; Mean absolute difference) at the 1^st^, 10^th^, 50^th^, 90^th^ and 99^th^ percentiles of the angular distributions (°) and the percentage of time above 30°, 60° and 90° for the 28 subjects during work, between the simplified reference analysis and the standard reference analysis

#### Simplified reference posture versus standard reference posture

**Fig. 3:**
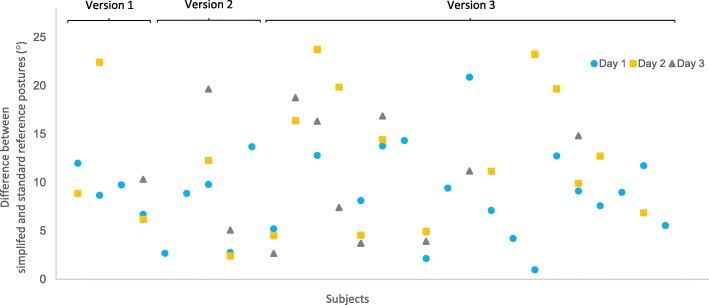
Individual differences (°) between the simplified reference posture and the standard reference posture on day 1 (), day 2 () and day 3 () for all 28 subjects

**Fig. 4:**
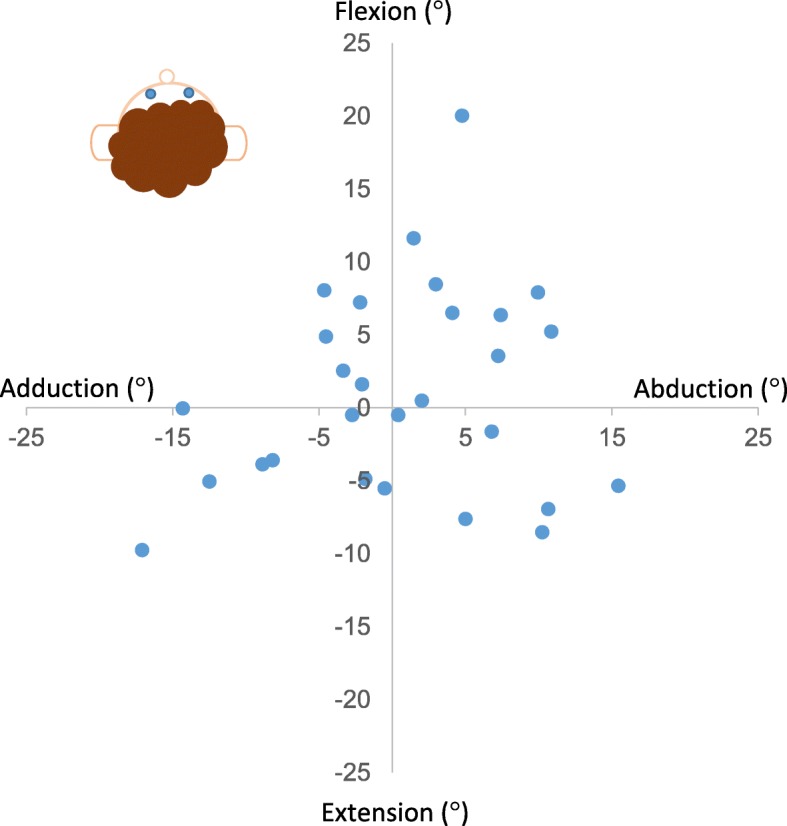
Individual arm position in the simplified reference posture () relative to the arm position in the standard reference posture on day 1 (day 2 for two subjects) for the 28 subjects

The differences (°) between the simplified reference posture and the standard reference posture on days 1, 2, and 3 for each subject are shown in Fig. 3. The group mean of the differences for day 1 (day 2 for two subjects) was 9° (range 1 – 21°). The individual arm position in the simplified reference posture relative to the arm position during the standard reference posture from day 1 (day 2 for two subjects) are shown in Fig. 4. They deviated in all directions (flexion, extension, adduction and/or abduction) without any obvious pattern.

#### Within-subject variation of simplified reference posture

The within-subject variation in the simplified reference posture was poor, with an ICC of 0.2 (Table 4). The repeatability coefficient was 16°.

**Table 4:** The within-subject variation of the simplified reference posture

Simplified reference posture			
Within-subject variation		Repeatability coefficient	ICC
SD	(95% CI)		
5.6	(3.7 – 7.5)	16	0.2

The within-subject variation (°; standard deviation (SD) and 95% confidence interval (95% CI)), the repeatability coefficient (°) and the intraclass correlation coefficient (ICC) of the simplified reference posture

### The protocol

No subjects were excluded due to an incorrect placement of the GC inclinometer. Nevertheless, we improved the protocol during the study. These changes appeared to make it easier for the subjects to follow, as the help needed decreased with improved versions of the protocol. An additional change (version 4) was made after the analyses, with instructions not to replace the GC inclinometer if it falls off.

The protocol includes three parts (see Additional file 1):

1. Starting the GC inclinometer.
2. Attaching the GC inclinometer to the upper arm.
3. Performing the simplified reference posture.

### Comparing different types of cleaning

Concerning self-recordings, the median upper arm velocity was higher in hotel housekeeping than in hotel housekeeping+ (82 vs 63 °/s; Table 5). There were no differences between self-recordings and researchers’ recordings of hotel housekeeping (Table 5).

**Table 5:** Group means of upper arm elevation and velocity during different types of cleaning

	Self-recordings during 3 days			Standard one-day recordings
	Hotel housekeeping (*n* = 9)	Hotel housekeeping+ (*n* = 11)	Office cleaning (*n* = 4)	Hotel housekeeping (*n* = 14)
	Mean (range)	Mean (range)	Mean (range)	Mean (range)
Elevation (°)				
50^th^	30 (25 – 36)	28 (22 – 35)	33 (29 – 38)	28 (21 – 38)
90^th^	65 (50 – 79)	62 (50 – 77)	64 (54 – 83)	61 (47 – 75)
Velocity (°/s)				
50^th^	82^a^ (53 – 114)	63^a^ (37 – 89)	56 (37 – 75)	92 (66 – 129)

Group means at the 50^th^ and 90^th^ percentiles of upper arm elevation (°) and the median generalised upper arm angular velocity (°/s) during different types of cleaning when using the standard reference posture as reference. (Data from the four men are excluded). The generalised angular velocity is not dependent on the reference posture. Hotel housekeeping = cleaning hotel rooms, hotel housekeeping+ = cleaning hotel rooms and other tasks such as cleaning corridors. The standard recordings were performed by researchers. Differences calculated by Kruskal-Wallis analysis of variance. Post hoc analysis with Mann-Whitney U-test

^a^*p* = 0.05

**Fig. 5:**
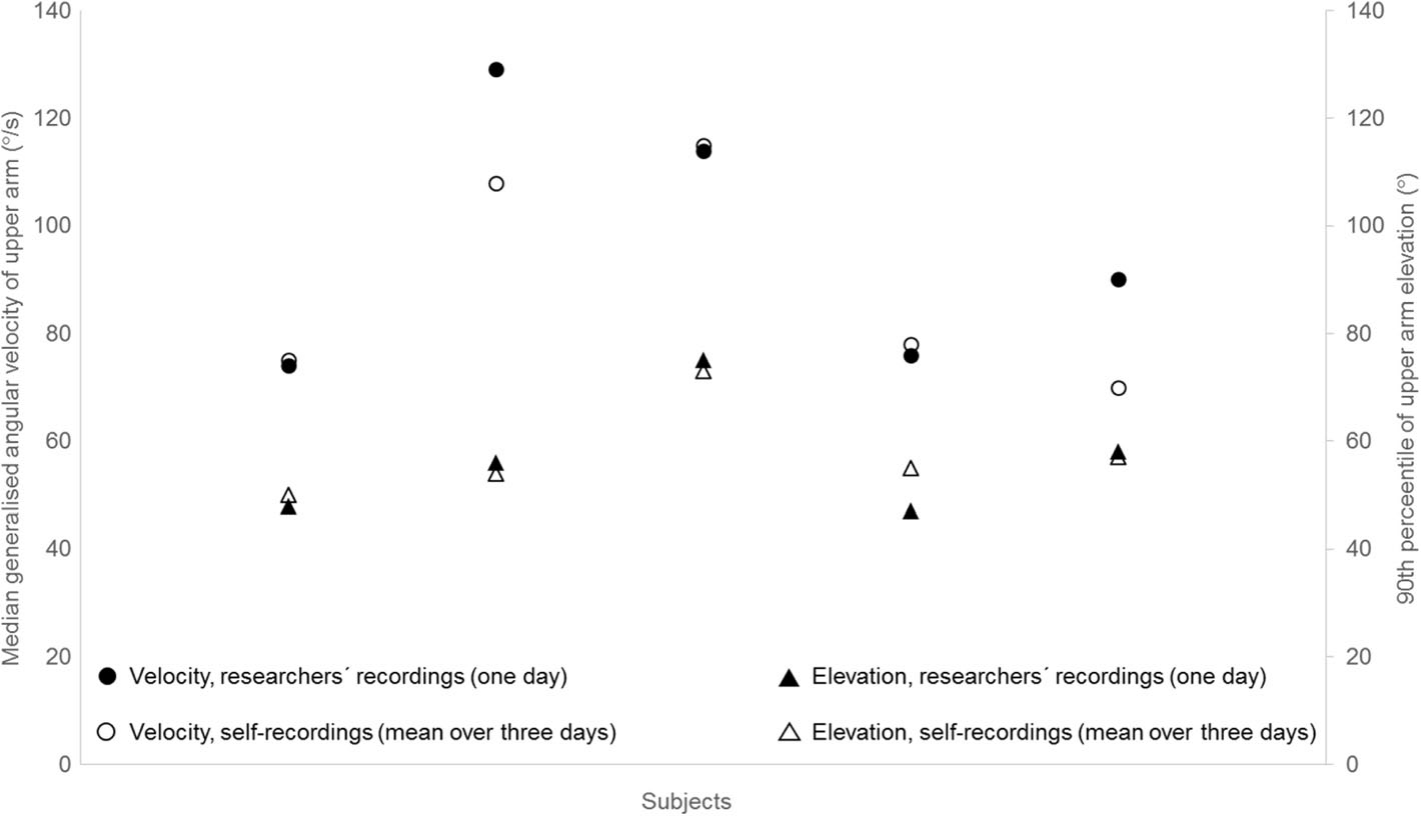
Upper arm elevation and the median generalised angular **v**elocity of the upper arm for the five subjects who participated in both the self-recordings and the researchers’ recordings. △ = upper arm elevation obtained with self-recording (using the standard reference posture). ○ = the median generalized upper arm velocity obtained with self-recording. ▲ = upper arm elevation obtained by the researchers’ recordings. ● = the median generalised upper arm velocity obtained by the researchers’ recordings

Five individuals participated in both the researchers’ recordings and the self-recordings. The 90^th^ percentile of upper arm elevation and the median generalised angular velocity for these individuals are shown in Figure 5. For them, the group mean difference for the 90^th^ percentile of upper arm elevation between the researchers’ recording on 1 day and the self-recording on several days, using the standard reference, was 1° (range -2 *–* 8°). The group mean difference for the upper arm velocity was -7 °/s (range -21 °/s *–* 2 °/s).

#### Within-subject variation in workload between days

**Fig. 6:**
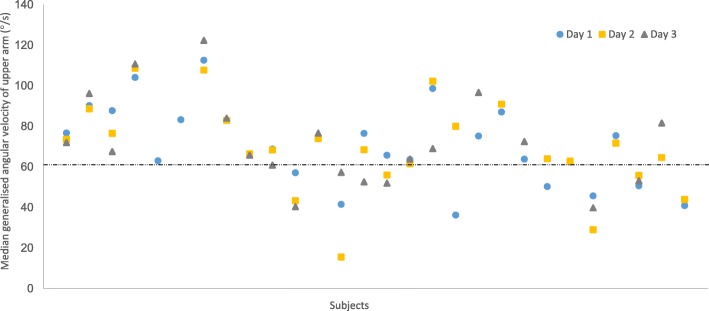
The median generalised angular velocity of the upper arm during work for the 28 subjects for day 1 (), day 2 () and day 3 (). The dashed line is the suggested action level for ergonomic workload

The repeatability coefficient for hotel housekeeping was 1.6° with an ICC of 0.98 for the 50th percentile of upper arm elevation (Table 6). Corresponding values for hotel housekeeping+ were 4.8° and 0.86, respectively. The individual variations in upper arm velocities during the different working days are shown in Fig. 6.

**Table 6:** The group means and the within-subject variations of upper arm elevation and velocity between working days

	Hotel housekeeping (*n* = 11)				Hotel housekeeping+ (*n* = 11)			
Percentile	Group mean (° or °/s)	Within-subject variation SD (95% CI)	Repeatability coefficient (° or °/s)	ICC	Group mean (° or °/s)	Within-subject variation SD (95% CI)	Repeatability coefficient (° or °/s)	ICC
50^th^ (°)	29	0.6 (0.3 – 0.9)	1.6	0.98	28	1.7 (0.9 – 2.5)	4.8	0.86
90^th^ (°)	64	1.5 (0.8 – 2.2)	4.1	0.97	62	4.4 (2.3 – 6.4)	12	0.80
Vel. (°/s)	81	4.7 (2.5 – 6.9)	13	0.93	63	12 (6.4 – 18)	33	0.66

The group mean, the within-subject variation (° or °/s; standard deviation (SD) and 95% confidence intervals (95% CI)), the repeatability coefficient (° or °/s) and the intraclass correlation coefficient (ICC) of upper arm elevations (°; 50th and 90th percentiles of the angular distribution) and median upper arm velocity (°/s; Vel.) between working days for 22 subjects. Self-recordings of hotel housekeeping and hotel housekeeping+. Hotel housekeeping = cleaning hotel rooms and hotel housekeeping+ = cleaning hotel rooms and other tasks such as cleaning corridors. The standard reference posture was used as reference

### The subjects’ perception of self-recording

The subjects’ perceptions are reported in Table 7. One subject answered “Bad” to one of the questions. All other answers were positive. Additionally, 87% of the subjects stated that the GC inclinometer had not interfered during work or leisure time during the three-day recording, and 96% were willing to wear the GC inclinometer again.

**Table 7:** Questionnaire responses after the self-recording

	Bad	Rather bad	Rather good	Good
How did you experience to wear the GC-inclinometer during several days?			4 (17%)	20 (83%)
How did you experience to sleep with the GC-inclinometer on?			5 (22%)	18 (78%)
How did you experience to shower with the sensor?			3 (15%)	17 (85%)
How did you experience to attach more plastic film?^a^	1 (7%)		1 (7%)	12 (86%)
How did you experience to perform the toe jumps and the reference position each morning?			1 (5%)	21 (95%)
How did you experience to fill in the diary?			7 (32%)	15 (68%)

Distribution of questionnaire responses from 24 subjects after self-recording of upper arm elevation and velocity during 3 days. The response rate (proportion within brackets) are given for the different options

^a^ eight subjects reported that this was not necessary

## Discussion

On group level, the recordings of upper arm elevation during work using the simplified reference posture were almost identical to the same recordings using the standard reference posture. The subjects were able to follow the instructions in the protocol and performed self-recording of upper arm elevations and velocities for several days.

### Simplified reference posture and standard reference posture

For recordings of arm elevations, it has been suggested that it is sufficient to attach an inclinometer with one of its axes aligned with the upper arm (humerus) without adopting a reference posture [15, 32]. However, since the humerus may not be parallel to the line of gravity, for example in subjects with voluminous upper arms (strong or obese), we believe that it is important to perform a reference posture to define 0° inclination. When using the standard reference posture the arm hangs out from the body (see Fig. 1a). Thus, this should be a minor problem. In the simplified reference posture the arm is closer to the body (see Fig. 1b). We therefore plotted the difference between the two reference postures from day 1 (Fig. 4) versus BMI. We saw no correlation, and do not suspect a major influence of BMI.

**Fig. 7:**
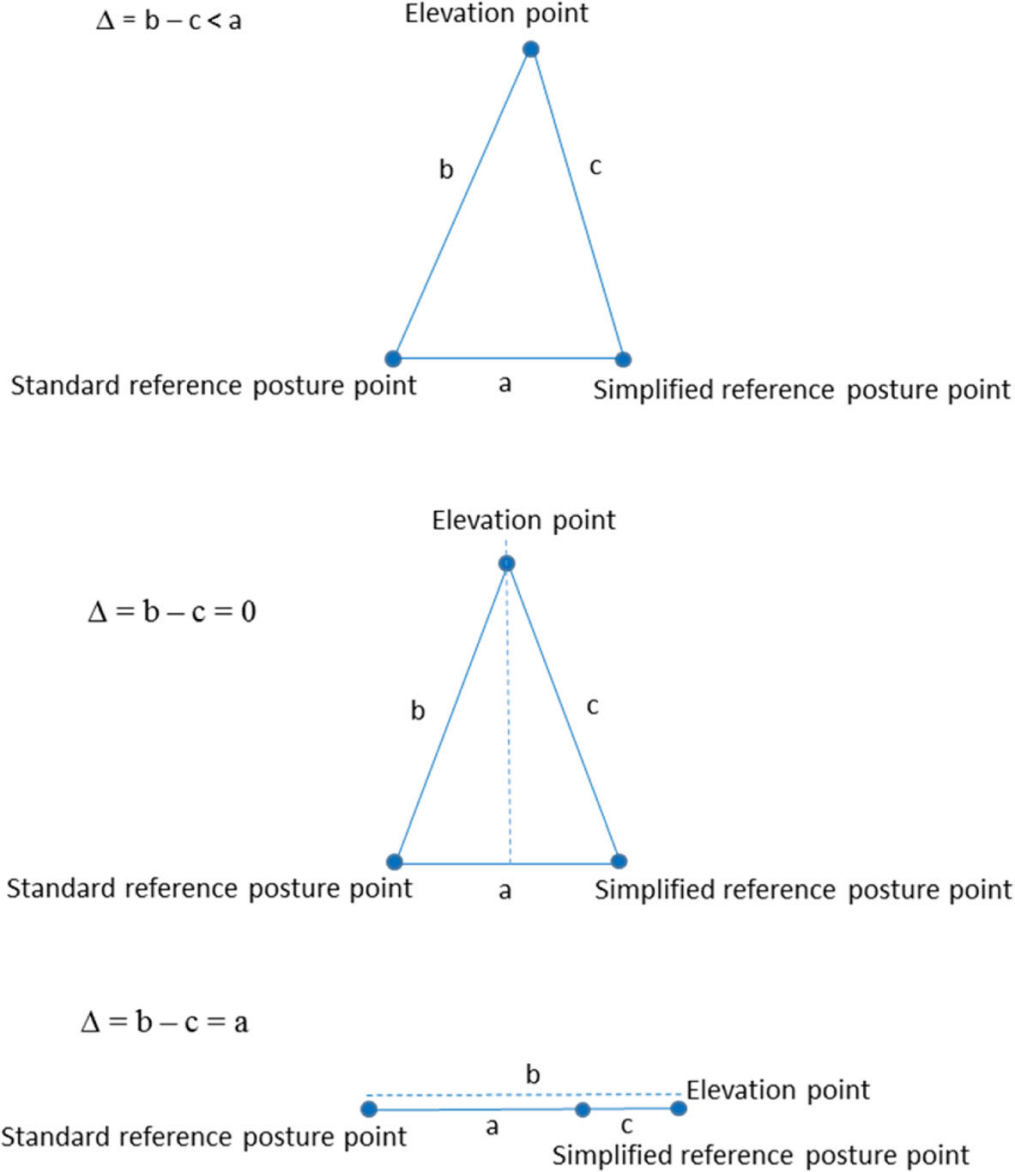
The triangle inequality may be used to explain that the average difference in upper arm elevation between the two analyses was lower than the difference between the two reference postures. The upper triangle illustrates the case that is likely to occur most of the work time. In that case the difference ∆ = b – c < a, where a is the difference between the two reference posture points, and b – c is the difference between the two analyses. In the (unusual) middle case ∆ = b – c = 0; the elevation point is equally far from the two reference posture points, and in the (unusual) lower case, ∆ = b – c = a, the elevation point is in line with and outside the two reference posture points, and the difference between the two reference analyses will be the same as the difference between the two reference postures. So ∆ is always less or equal to the difference between the two reference posture points

For each individual, the difference in upper arm elevation during work between the two analyses was lower than the difference between the two references, and may be explained by the triangle inequality (see Fig. 7). The distance between the two reference points can be seen as the length of one side of a triangle (a). The distance between one of the reference points and a specific elevation point during work can then be seen as the length of a second side of the triangle (b), while the distance between the other reference point and the same specific elevation point can be seen as the length of the third side of the triangle (c). Thus, as the length of one side in a triangle is less than the difference (∆) of the lengths of the two other sides, the difference between the two reference analyses will be less than the difference between the two references (∆ = b – c < a). For an elevation point that is equally far from the two reference points, the triangle becomes isosceles and the difference between the two reference analyses will be zero (∆ = b – c = 0). If the elevation point is in line with the two references, the triangle becomes a line and the difference between the two reference analyses will be the same as the difference between the two references (∆ = b – c = a). In this study, the difference during work was never more than 10°, while the difference between the two references was up to 21°. In addition, the group mean difference during work was as low as 0.2° (range -7 *–* 10°). We therefore consider, on group level, the simplified reference posture sufficient for recording of elevations of the upper arm, given that it is the same work tasks and a low degree of freedom in work performance for all individuals [33]. In the current group of cleaners, the simplified reference posture deviated from the standard reference in a uniform pattern (i.e. in all directions, see Fig. 4). Consequently, deviations during work were balanced on group level. However, this may not be the case in other populations. A non-uniform deviation pattern will introduce a systematic error. Concerning upper arm velocity, the self-recording method can be used on individual level, as this measure is not dependent on the reference.

#### Within-subject variation of simplified reference posture

In a previous study of natural head posture recorded with inclinometer, the individual overall variability (standard deviation) was 1.6° [34]. In our study, the standard deviation of the within-subject variation was 5.6° for the simplified reference posture, i.e. somewhat higher. We speculate that this difference may be because it is more difficult to repeat an arm posture (without support) than a head posture, as in the latter the sight angle serves as a reference. The repeatability coefficient for the simplified reference posture was 16°. Thus, in 95% of measurements, the absolute difference between two simplified reference measurements on one subject is not expected to exceed 16°. Therefore, a recording of upper arm elevations analysed with the simplified reference posture at only one occasion should be interpreted with some caution.

### The protocol and the subjects’ perceptions of self-recording

The protocol was continuously improved during the study. Thereby, the problems that occurred during the study were resolved. Most importantly, if the GC inclinometer falls off it should not be replaced. Further, toe jumps are performed before *and* after the simplified reference posture. We believe that version 4 is easy to use. Still, for subjects that do not speak Swedish or English, one might consider to translate it into the language in question.

According to the questionnaire which the subjects answered after the study, all but one of the subjects were positive to self-recordings of upper arm elevations and velocities. Only one person answered “Bad” to the question “How did you experience to put on more plastic film?” Since eight subjects reported that it had not been necessary, we think this negative answer was due to language barriers, and this subject also meant that it had not been necessary.

### Risk of musculoskeletal disorders among cleaning staff

Our research group has performed technical measurements of upper arm elevations and velocities for about thirty years in about sixty different occupations. Most of these occupational groups have also been clinically examined using the standardised Health Surveillance in Adverse Ergonomics Conditions (HECO) method [35, 36] which quantifies the prevalence of WMSDs and diagnoses of the neck and upper extremities. Exposure-response relationships were obtained by compiling the data from the technical measurements and the clinical examinations, and we found strong associations between upper arm velocity and several diagnoses [2]. Based on this knowledge, we have recently proposed action levels for the prevention of WMSDs. The proposed action level for the median generalised angular velocity is 60 °/s [37]. This is well in line with the findings in a recent study by Dalbøge et al., where it was indicated that a median generalised angular velocity of the upper arm below 45 °/s was safe [38]. Based on previous studies [2, 39–42], we have proposed an action level of 60° for the 90^th^ percentile of upper arm elevation. The action level for elevation was exceeded in office cleaning, while the action levels for both elevation and angular velocity were exceeded in hotel housekeeping (both self-recordings and researchers’ recordings) and hotel housekeeping+, indicating the need for preventive actions. Hence, it was highly relevant to test the self-recording method among cleaners.

#### Within-subject variation of workload between working days

The within-subject variation in upper arm elevation and velocity between working days in hotel housekeeping was low. This indicates that the work is monotonous and repetitive. The between days variation differed between hotel housekeeping + and hotel housekeeping, and one explanation could be that there were additional and more varied work tasks in hotel housekeeping+, such as for example cleaning corridors, conference rooms and pool areas.

### Methodological considerations

To the best of our knowledge, this is the first time self-recordings have been made of upper arm elevation and velocity. This required a protocol explaining how to perform the self-recording. A strength of the study was that the protocol was tested and improved in an occupation with a high proportion of immigrants. Even if the subjects spoke poor Swedish and English, they were able to perform self-recordings. This indicates that the protocol is easy to follow and may be used by most employees. A weakness is that we did not improve the protocol systematically and did not evaluate the different steps of improvements in a systematic manner. Instead, we made changes in the protocol based on how comfortable and secure the subjects appeared to be when they attached the GC inclinometer and performed the simplified reference posture. On a visual inspection of Fig. 2 we did not see any improvement concerning the individual differences between the two analyses. Thus, we do not think that different versions of the protocol impacted on our data.

Considering recordings of upper arm elevation, we judge a difference of 5° to be clinically relevant. Prior to the study we did not know the distribution of the differences between the analyses with the two different reference postures. As this was about 5° for both the 50th and the 90th percentiles we would have needed 11 subjects to be able to detect a 5° difference between the two analyses with an 80% power. As 28 cleaners were included, we could detect a difference of 3°.

## Conclusions

The small difference between the simplified reference analysis and the standard reference analysis indicates that recordings performed by employees themselves are comparable, on group level, with those performed by researchers. The subjects in this study were able to perform self-recording of upper arm elevations and velocities using the protocol provided. The simplified reference posture is sufficient on group level, with the assumption that it is the same work tasks and a high similarity in work performance for all individuals. The self-recording method can be used at an individual level for recording of upper arm velocity. Self-recording could increase the use of technical methods when performing risk assessments and, in combination with action levels for the prevention of WMSDs, increase the accuracy of risk assessments. In addition, self-recording in combination with action levels would provide employers with a method of assessing the risk of developing WMSDs among employees, which would be an important improvement of prevention. Hotel cleaning implies a high risk of musculoskeletal disorders due to a high upper arm velocity.

## Additional files

Additional file 1: The protocol (version 4) “Instructions for self-recording of upper arm elevation and velocity”. (DOCX 1071 kb)

Additional file 2: Datasets of upper arm elevation using simplified and standard reference postures. (XLSX 26 kb)

## Abbreviations

GC inclinometer: Gulf Coast triaxial accelerometer X16-mini
WMSDs: Work-related musculoskeletal disorders
