# Supplementary material for: Self-recordings of upper arm elevation during cleaning – comparison between analyses using a simplified reference posture and a standard reference posture
Source: BMC Musculoskelet Disord. 2018 Nov 15;19:402. doi: 10.1186/s12891-018-2328-8 (PMC6238373; doi:10.1186/s12891-018-2328-8)
Supplement: Supplementary file 1 — The protocol (version 4) “Instructions for self-recording of upper arm elevation and velocity”. (DOCX 1071 kb) [file 12891_2018_2328_MOESM1_ESM.docx]

**Instructions for self-recording of upper arm elevation and velocity**

Please read these instructions carefully before you put the sensor on.

1. Start the sensor


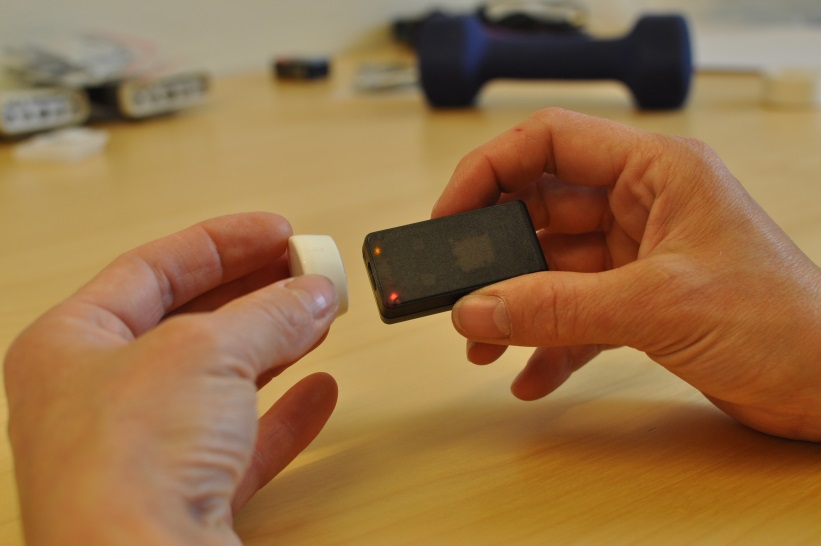


Touch the USB connector on the magnet. A yellow and a red lamp start to blink. Remove the magnet immediately. Make sure the sensor is operating: the yellow lamp should be blinking all the time, and the red lamp now and then.


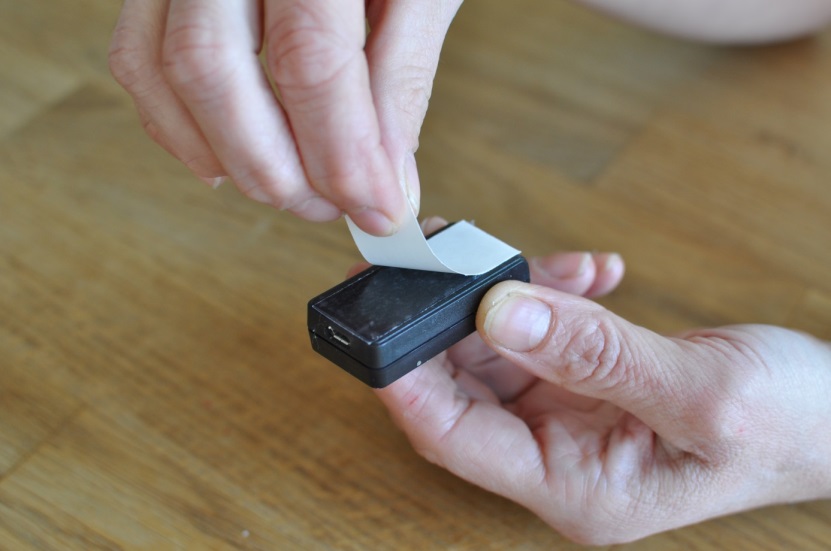


2.

Attach the sensor to your arm as shown in the picture.

Remove the white paper strip from the back of the sensor.

**
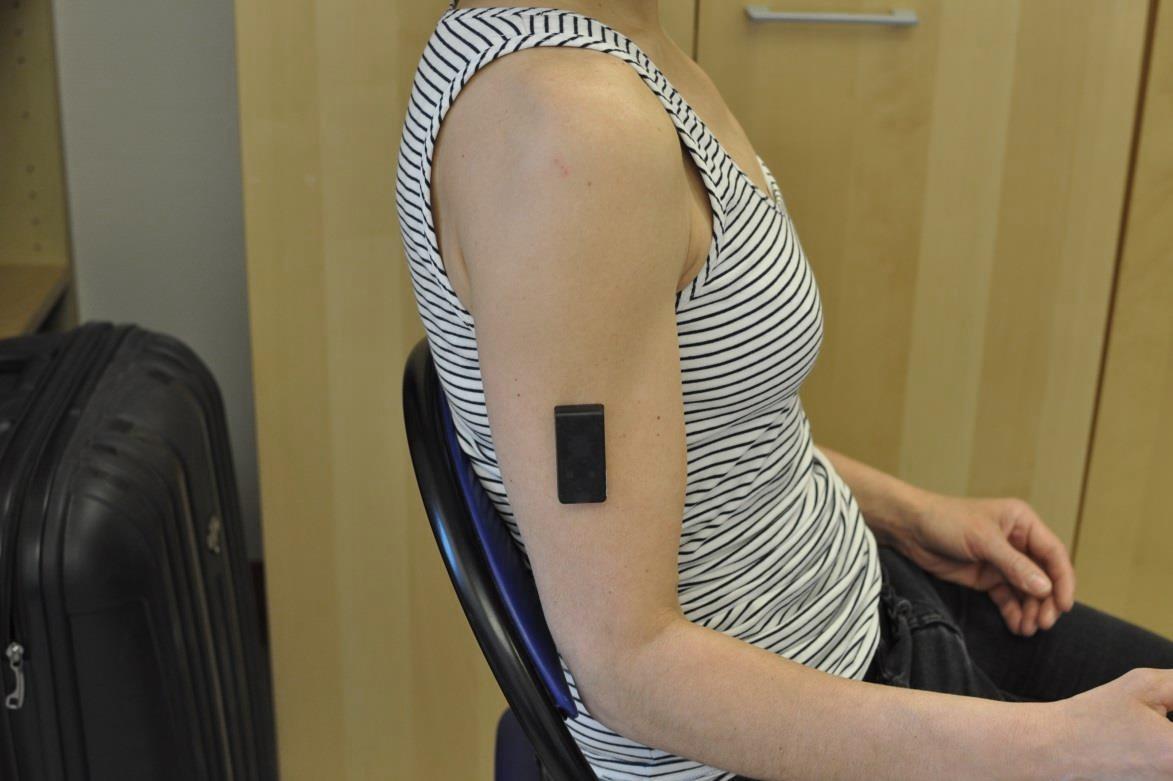
**

3. Attach it


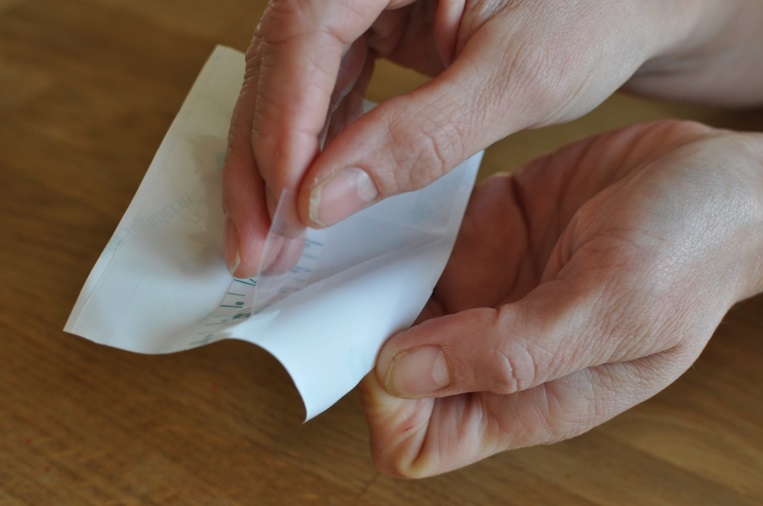

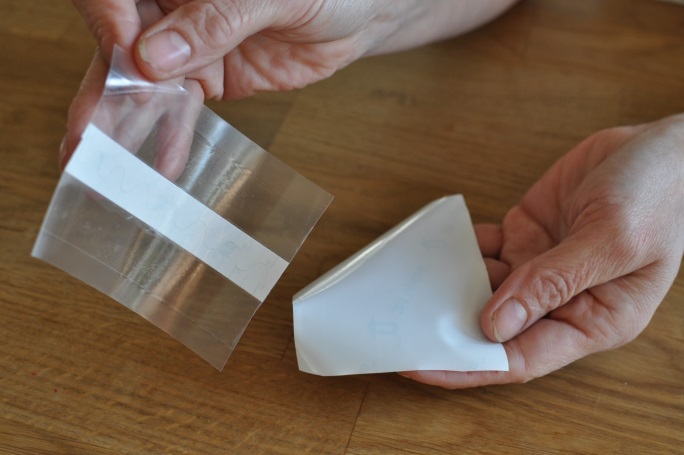


Remove the white backing from the clear plastic film.

4.

5.


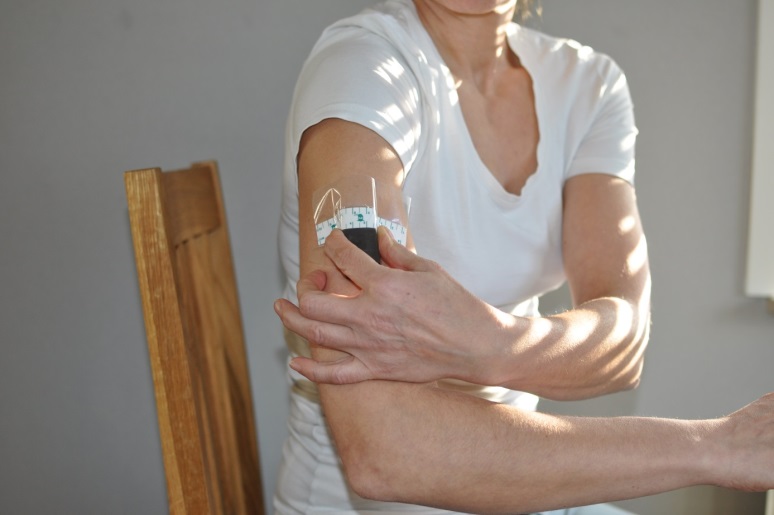
**
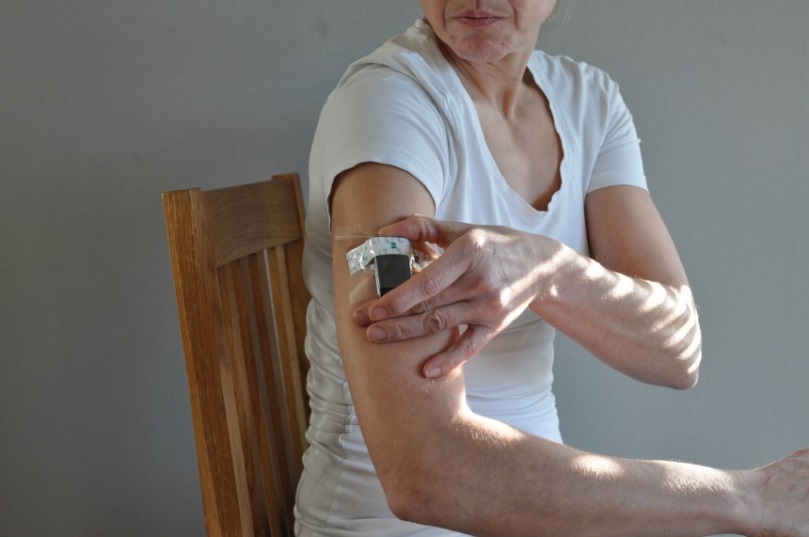
**

1. Place the plastic film over the sensor.

2. Press the plastic film firmly along the sides and ends of the sensor.

6.

**
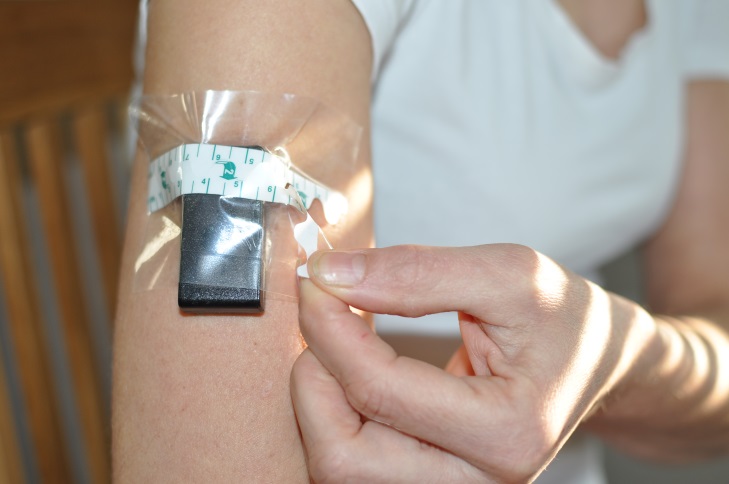
**
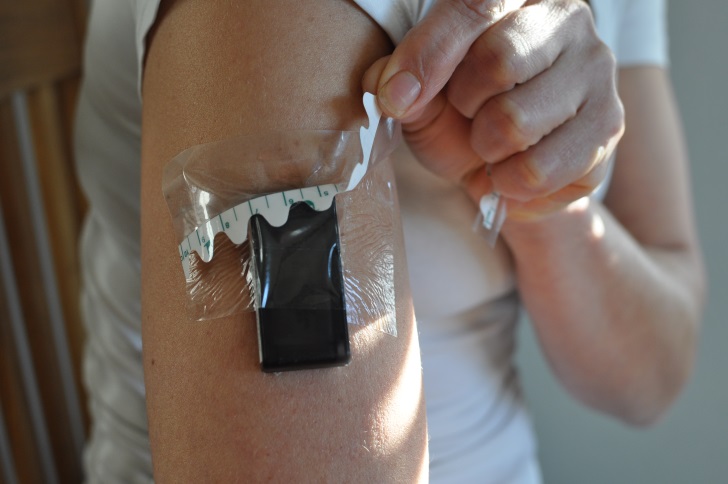


Grab one of the white strips on the film to remove one half of the covering plastic film. Then remove the other half, leaving only a thin plastic film.


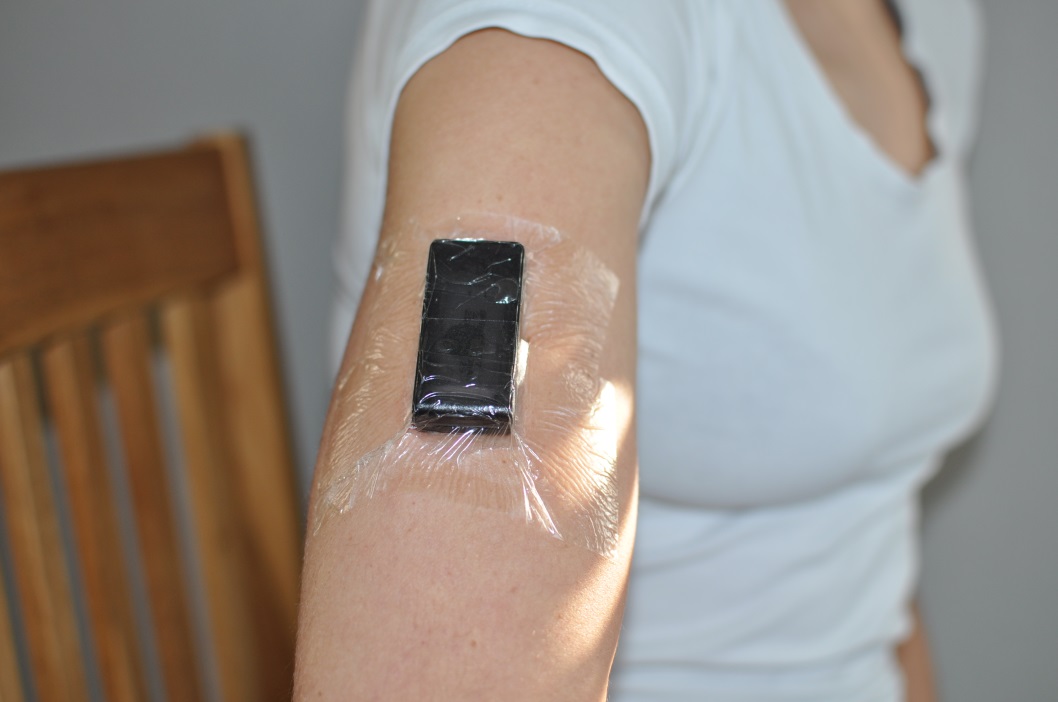


7.

The thin plastic film should cover the whole of the sensor and part of the arm. Please, use more plastic films if you have not managed to cover the area shown in the picture.


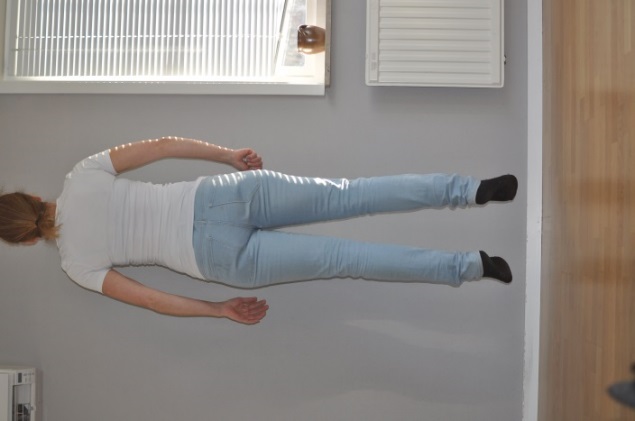


You will now perform **5 toe jumps**. Jump up and down 5 times.

9. Zero position

8. Toe jumps


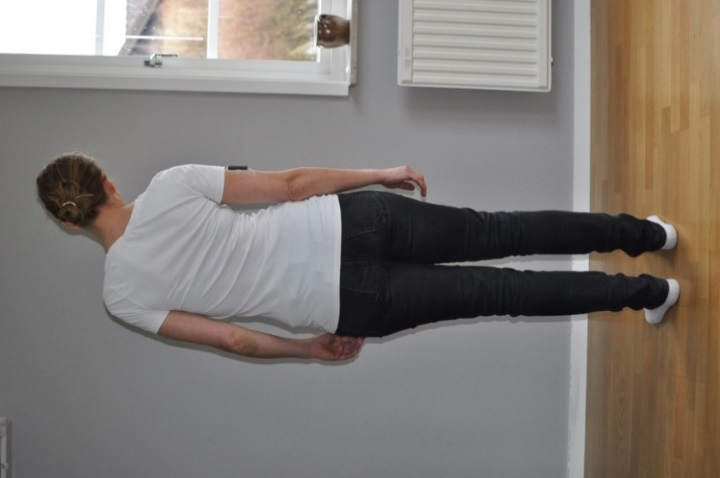

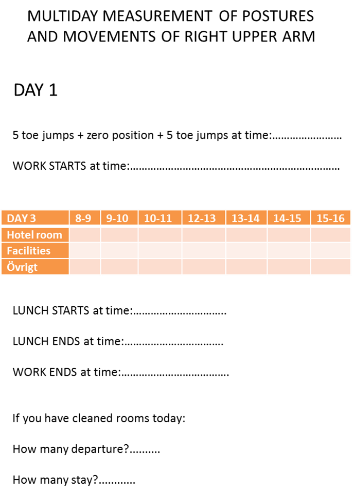


Immediately after the toe jumps, you should stand in **the zero position.** Follow these instructions carefully:

1. Lean to the right and hold your arm by your side, as shown in the picture. Extend the elbow.

**Hold this position for 20 seconds.**

2. **IMPORTANT** - Write down the exact time (hh:mm:ss) in the protocol under day 1.


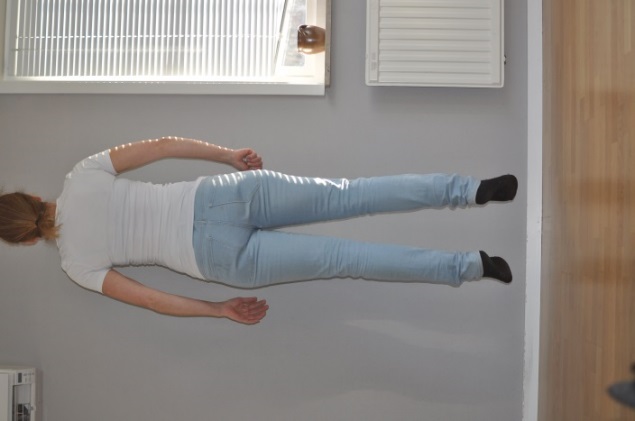


Perform **5 toe jumps** once again. Jump up and down 5 times.

10. Toe jumps again

**You are now ready to start work.**

Please note the time you start and stop working, and the time you start and stop lunch and breaks, in the form supplied. The sensor should remain on your arm for the whole of the study period. Do not remove it to shower or when you go to bed.

**If the sensor falls off, please note the date and time here:**

**…………/………(day/month)**

**………:…………:……….(hh:mm:ss)**

You should not replace it.

If you **feel any pain**, or if your **skin starts to itch,** or **turns red** around the sensor**, remove it immediately**. **Please note the date and time here:**

**…………/………(day/month)**

**………:…………:……….(hh:mm:ss)**

Please feel free to give me a call or send me a text message if you have any questions.

Kindly,

**Forename Surname**, Institution, **phone xxxxxxxx, mobile phone xxxxxxxx**
